# Correction: Functionally diverse human T cells recognize non-microbial antigens presented by MR1

**DOI:** 10.7554/eLife.29743

**Published:** 2017-06-20

**Authors:** Marco Lepore, Artem Kalinichenko, Salvatore Calogero, Pavanish Kumar, Bhairav Paleja, Mathias Schmaler, Vipin Narang, Francesca Zolezzi, Michael Poidinger, Lucia Mori, Gennaro De Libero

Lepore M, Kalinichenko A, Calogero S, Kumar P, Paleja B, Schmaler M, Narang V, Zolezzi F, Poidinger M, Mori L, De Libero G. 2017. Functionally diverse human T cells recognize non-microbial antigens presented by MR1. *eLife* **6**:e24476. doi: 10.7554/eLife.24476.

Published 18, May 2017

In Table 1, the TCRα V gene segment of the clone DGA28 has been indicated by mistake as TRAV21. The correct attribution is TRAV25.

The corrected Table 1 is shown here.

**Table 1.** Phenotype and TCR gene usage of selected MR1-reactive T cell clones.

Clone	CD4	CD8α	TCRα	TCRβ	CD161
DGB129	-	+	TRAV29	TRBV12-4	-
DGB70	-	-	TRAV5	TRBV28	-
DGA28	-	+	TRAV25	TRBV29-1	+
DGA4	-	-	TRAV1-2	ND	+
JMA	-	+	TRAV27	TRBV25-1	-
TC5A87	-	+	TRAV13-1	TRBV25-1	-
CH9A3	-	+	TRAV24	TRBV5-5	-

ND, not determined.

In Figure 8–figure supplement 1, the symbols in Panel B were wrongly assigned.

The corrected Figure 8–figure supplement 1 is shown here.

**Figure 1:**
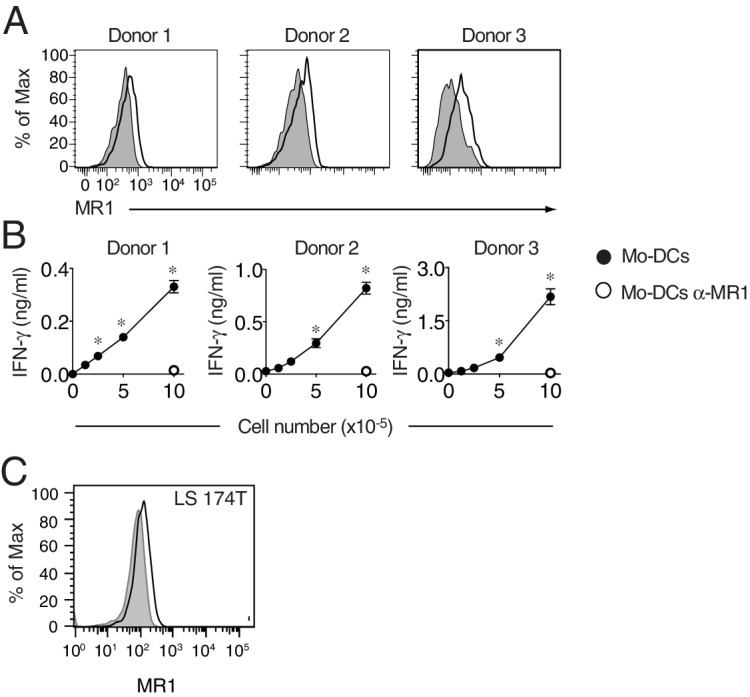

The originally published Figure 8–figure supplement 1 is shown for reference

**Figure 2:**
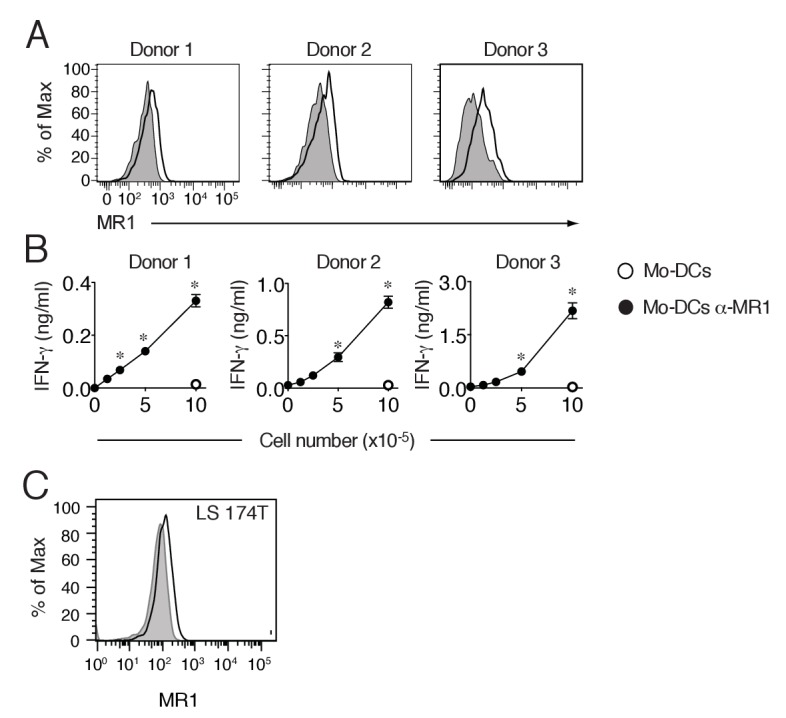

The article has been corrected accordingly.

