# Supplementary material for: Functionally diverse human T cells recognize non-microbial antigens presented by MR1
Source: eLife. 2017 May 18;6:e24476. doi: 10.7554/eLife.24476 (PMC5459576; doi:10.7554/eLife.24476)
Supplement: Supplementary file 1. — DOI: http://dx.doi.org/10.7554/eLife.24476.016 [file elife-24476-supp1.docx]

**Supplementary file 1**

| **Genes modulated in activated *vs.* resting MR1T cell clones.** | | | |
| --- | --- | --- | --- |
|  |  |  |  |
| **DGB129** |  | **DGB70** |  |
| **Gene ID** | **Log_2_ fold change** | **Gene ID** | **Log_2_ fold change** |
| *NKD1* | -6.446 | *KIAA1549L* | -5.855 |
| *FCRL6* | -6.162 | *CTD-2206N4.4* | -5.297 |
| *RP1-193H18.2* | -5.975 | *SLC17A3* | -5.144 |
| *KIAA1549L* | -5.832 | *RTKN2* | -5.111 |
| *MAP2K6* | -5.628 | *GATA6* | -5.100 |
| *CDH23* | -5.485 | *SLC14A1* | -5.025 |
| *LINC01057* | -5.481 | *PLXDC1* | -4.950 |
| *PER3* | -5.481 | *RP11-730K11.1* | -4.833 |
| *LY9* | -5.436 | *CDCP1* | -4.736 |
| *SLAMF8* | -5.259 | *DPEP2* | -4.693 |
| *PFN1P7* | -5.190 | *C12orf42* | -4.673 |
| *MPP7* | -5.083 | *RP11-763B22.3* | -4.648 |
| *GATA6* | -4.676 | *NKD1* | -4.570 |
| *PLXDC1* | -4.671 | *NEURL1B* | -4.559 |
| *AP001055.1* | -4.631 | *PER3* | -4.371 |
| *C1orf204* | -4.593 | *FCRL6* | -4.292 |
| *ZNF683* | -4.504 | *RP1-193H18.2* | -4.287 |
| *ADAMTS17* | -4.451 | *RP11-190A12.8* | -4.277 |
| *MEGF6* | -4.414 | *RP11-37C7.3* | -4.274 |
| *RP11-401P9.4* | -4.379 | *MAP2K6* | -4.248 |
| *CHI3L2* | -4.334 | *SLFN14* | -4.231 |
| *DDX4* | -4.334 | *SLAMF8* | -4.224 |
| *RP11-190A12.8* | -4.313 | *CACNA1I* | -4.216 |
| *CEBPD* | -4.299 | *RP11-147L13.8* | -4.163 |
| *CTD-2206N4.4* | -4.239 | *SYNGR1* | -4.144 |
| *RASGRP2* | -4.190 | *CTD-3092A11.2* | -4.046 |
| *ST8SIA6* | -4.190 | *CPNE7* | -4.015 |
| *ADRB2* | -4.109 | *MGAT3* | -3.974 |
| *SLFN14* | -4.099 | *AMY1A* | -3.936 |
| *C12orf42* | -4.046 | *TCF7* | -3.908 |
| *RP11-747H7.3* | -4.039 | *C1orf204* | -3.856 |
| *HSPA6* | -4.029 | *RP11-401P9.4* | -3.850 |
| *TNFSF13B* | -4.008 | *WHAMMP3* | -3.827 |
| *DPEP2* | -3.951 | *RP11-713M15.2* | -3.815 |
| *GSTM5* | -3.911 | *LY9* | -3.751 |
| *EGR3* | -3.904 | *AMY1B* | -3.722 |
| *FCGBP* | -3.859 | *RP11-1094M14.12* | -3.681 |
| *RP11-1094M14.12* | -3.824 | *SCML4* | -3.573 |
| *RP11-147L13.8* | -3.815 | *PBK* | -3.570 |
| *GAS6* | -3.787 | *HSPA6* | -3.537 |
| *RP11-730K11.1* | -3.705 | *CTC-303L1.1* | -3.515 |
| *ZNF541* | -3.702 | *FCGBP* | -3.496 |
| *PLCH2* | -3.691 | *CTD-3092A11.1* | -3.490 |
| *GSTM2* | -3.677 | *TROAP* | -3.480 |
| *CATSPERB* | -3.635 | *CABP4* | -3.439 |
| *ANK3* | -3.624 | *PNMA3* | -3.435 |
| *CTC-523E23.11* | -3.580 | *TTLL1* | -3.428 |
| *TC2N* | -3.565 | *RP11-632K20.7* | -3.426 |
| *AIM1L* | -3.539 | *KIF14* | -3.420 |
| *LILRP2* | -3.527 | *RHOU* | -3.393 |
| *TCF7* | -3.497 | *MEGF6* | -3.386 |
| *KCNA2* | -3.486 | *RPS6KA2* | -3.368 |
| *MGAT3* | -3.482 | *GAS6* | -3.356 |
| *NCAM1* | -3.468 | *KIF18B* | -3.342 |
| *CUBN* | -3.465 | *RP11-2B6.2* | -3.331 |
| *ST8SIA1* | -3.465 | *RP11-666A1.3* | -3.312 |
| *CTC-303L1.1* | -3.455 | *MPZL2* | -3.310 |
| *RPS6KA2* | -3.438 | *ASPM* | -3.299 |
| *ABLIM1* | -3.432 | *MIRLET7DHG* | -3.282 |
| *CTA-373H7.7* | -3.404 | *NEK2* | -3.277 |
| *KLHDC1* | -3.386 | *ZNF33B* | -3.276 |
| *MIAT* | -3.378 | *IL7R* | -3.265 |
| *DAPK2* | -3.346 | *CCDC141* | -3.248 |
| *CLU* | -3.330 | *HJURP* | -3.243 |
| *LINC00514* | -3.311 | *DDX4* | -3.235 |
| *AC131056.3* | -3.291 | *CD40LG* | -3.220 |
| *AL161784.1* | -3.289 | *ZBTB16* | -3.219 |
| *SLC14A1* | -3.284 | *ADRB2* | -3.213 |
| *ABCA7* | -3.277 | *TTC21A* | -3.192 |
| *RNASE6* | -3.270 | *ZNF683* | -3.184 |
| *NEURL1B* | -3.264 | *AURKB* | -3.165 |
| *AUTS2* | -3.240 | *KIF20A* | -3.156 |
| *SLC9A9* | -3.224 | *GSTM5* | -3.144 |
| *TNNT3* | -3.217 | *TRPM2* | -3.142 |
| *SCML4* | -3.201 | *SORL1* | -3.136 |
| *ATXN7L1* | -3.200 | *TNFRSF10A* | -3.133 |
| *CTC-246B18.8* | -3.180 | *ZCCHC18* | -3.108 |
| *TPM2* | -3.173 | *RP11-747H7.3* | -3.102 |
| *PCSK4* | -3.157 | *GRB10* | -3.095 |
| *CTC-523E23.5* | -3.147 | *GNAO1* | -3.094 |
| *STOM* | -3.145 | *TTK* | -3.083 |
| *CYB561* | -3.138 | *RP3-328E19.4* | -3.060 |
| *GNAO1* | -3.136 | *PIF1* | -3.052 |
| *CECR1* | -3.135 | *GSTM2* | -3.020 |
| *NR1D2* | -3.132 | *ANKEF1* | -3.001 |
| *NUAK2* | -3.132 | *AKAP7* | -2.978 |
| *SIGIRR* | -3.126 | *CENPF* | -2.970 |
| *RP11-713M15.2* | -3.125 | *CDC20* | -2.958 |
| *RGS18* | -3.123 | *DEPDC1* | -2.952 |
| *COLGALT2* | -3.118 | *RASGRP2* | -2.951 |
| *KIF14* | -3.103 | *RP11-18H21.1* | -2.945 |
| *TMEM204* | -3.093 | *KIT* | -2.926 |
| *GRB10* | -3.072 | *PTPRN2* | -2.921 |
| *ZBP1* | -3.059 | *GSTM1* | -2.920 |
| *RP11-797H7.1* | -3.057 | *AC109826.1* | -2.914 |
| *AHNAK* | -3.051 | *KIF15* | -2.908 |
| *C11orf21* | -3.046 | *BCORP1* | -2.902 |
| *GSTM1* | -3.046 | *PLXND1* | -2.898 |
| *TTC22* | -3.035 | *MKI67* | -2.897 |
| *SCUBE1* | -3.029 | *CTAGE7P* | -2.885 |
| *TRBV5-4* | -3.029 | *MPP7* | -2.885 |
| *CD101* | -3.028 | *TC2N* | -2.868 |
| *AQP3* | -3.012 | *KCNQ1* | -2.863 |
| *TMOD2* | -3.007 | *TMOD2* | -2.861 |
| *DISC1* | -2.998 | *ZMYND10* | -2.861 |
| *PFN1P6* | -2.995 | *HCG27* | -2.852 |
| *RP11-744H18.1* | -2.992 | *AC079922.3* | -2.841 |
| *LINGO3* | -2.991 | *AMY1C* | -2.833 |
| *RP1-151B14.6* | -2.989 | *OSER1-AS1* | -2.824 |
| *CCL3L3* | -2.987 | *NR1D2* | -2.820 |
| *GSTM4* | -2.969 | *RP11-89F3.2* | -2.805 |
| *SORL1* | -2.947 | *NEIL3* | -2.785 |
| *CTD-2207L17.1* | -2.942 | *KIAA0825* | -2.772 |
| *LINC00861* | -2.942 | *XXbac-BPG299F13.14* | -2.766 |
| *RP11-111E14.1* | -2.935 | *DLGAP5* | -2.760 |
| *B3GALT2* | -2.920 | *RP11-1149O23.2* | -2.750 |
| *IL7R* | -2.920 | *HMMR* | -2.738 |
| *AC007952.6* | -2.919 | *BIRC5* | -2.733 |
| *TSPAN32* | -2.906 | *IFNL1* | -2.732 |
| *TRAM2* | -2.905 | *ANLN* | -2.726 |
| *XXbac-BPG299F13.14* | -2.896 | *CDCA8* | -2.711 |
| *LRRC37A9P* | -2.888 | *TOP2A* | -2.684 |
| *KCNQ1* | -2.880 | *RP11-744H18.1* | -2.683 |
| *IRGM* | -2.877 | *MIR24-2* | -2.673 |
| *LINC00426* | -2.873 | *RCBTB2* | -2.652 |
| *ASPM* | -2.868 | *TNNT3* | -2.651 |
| *KIAA0825* | -2.856 | *BSN* | -2.645 |
| *ZFR2* | -2.854 | *S100A4* | -2.640 |
| *RHOU* | -2.846 | *RP4-758J18.13* | -2.640 |
| *AC009133.17* | -2.842 | *CHRFAM7A* | -2.632 |
| *TNFRSF10A* | -2.842 | *ZNF449* | -2.622 |
| *AC015849.16* | -2.829 | *VWCE* | -2.622 |
| *LINC00537* | -2.822 | *AC000032.2* | -2.618 |
| *IQGAP3* | -2.810 | *RNASE6* | -2.611 |
| *RP11-204N11.1* | -2.807 | *TRIP13* | -2.608 |
| *AC092316.1* | -2.800 | *PAQR8* | -2.607 |
| *MORN3* | -2.795 | *AC131056.3* | -2.596 |
| *EFHC1* | -2.788 | *IQGAP3* | -2.593 |
| *BCORP1* | -2.782 | *RP11-47I22.3* | -2.586 |
| *TMEM116* | -2.778 | *PRR11* | -2.584 |
| *RP11-403I13.7* | -2.776 | *GLB1L2* | -2.580 |
| *PLXNA4* | -2.767 | *LINC00861* | -2.563 |
| *TRANK1* | -2.765 | *AC087645.1* | -2.561 |
| *ZNF449* | -2.757 | *KIF23* | -2.548 |
| *HCG27* | -2.753 | *PLCH2* | -2.546 |
| *RASGRF2* | -2.751 | *CATSPERB* | -2.540 |
| *C17orf66* | -2.737 | *ZFHX3* | -2.537 |
| *DNM1P47* | -2.734 | *LEF1* | -2.536 |
| *CFH* | -2.723 | *LILRP2* | -2.526 |
| *AMY1B* | -2.723 | *RIMBP3C* | -2.518 |
| *RP11-473M20.5* | -2.721 | *C11orf21* | -2.518 |
| *IRS1* | -2.707 | *ABCA7* | -2.515 |
| *AC079922.3* | -2.692 | *RP11-666A1.5* | -2.509 |
| *SYNE2* | -2.681 | *CIT* | -2.503 |
| *SYNGR1* | -2.679 | *TSPAN32* | -2.482 |
| *TXK* | -2.679 | *E2F7* | -2.480 |
| *FRAT1* | -2.672 | *NT5M* | -2.474 |
| *CTD-3092A11.2* | -2.661 | *RP11-763B22.4* | -2.472 |
| *DHRS12* | -2.660 | *PROK2* | -2.467 |
| *GSN* | -2.660 | *MIR142* | -2.466 |
| *PSRC1* | -2.660 | *LTB4R* | -2.464 |
| *PLA2G4B* | -2.658 | *AP000351.10* | -2.464 |
| *RNU1-143P* | -2.654 | *IRGM* | -2.455 |
| *RP3-328E19.4* | -2.651 | *MGLL* | -2.447 |
| *RSAD2* | -2.641 | *C7orf41* | -2.444 |
| *FAIM3* | -2.641 | *CDH23* | -2.442 |
| *LINC00086* | -2.641 | *RP11-473M20.5* | -2.425 |
| *MYO1F* | -2.638 | *RASGEF1A* | -2.417 |
| *ZNF33B* | -2.635 | *GTSE1* | -2.412 |
| *RASGRP3* | -2.631 | *RIMBP3B* | -2.406 |
| *STXBP5-AS1* | -2.628 | *CTD-2015B23.2* | -2.400 |
| *RP11-160E2.11* | -2.620 | *C5orf63* | -2.400 |
| *PLCD1* | -2.605 | *NMT2* | -2.372 |
| *ITGA1* | -2.605 | *CDCA5* | -2.370 |
| *KIAA1107* | -2.599 | *AQP3* | -2.363 |
| *PDGFD* | -2.599 | *LINC00892* | -2.361 |
| *ADCY4* | -2.598 | *SCUBE1* | -2.346 |
| *TROAP* | -2.597 | *Z85986.1* | -2.345 |
| *IL24* | -2.596 | *AIM1L* | -2.340 |
| *SLC22A18* | -2.593 | *ADAM23* | -2.337 |
| *ADARB2* | -2.586 | *LINC01057* | -2.331 |
| *ZCCHC18* | -2.586 | *CCNB2* | -2.328 |
| *RP11-799D4.4* | -2.583 | *RP11-539L10.2* | -2.326 |
| *AMZ1* | -2.582 | *RP4-738P11.4* | -2.326 |
| *C1QTNF6* | -2.579 | *IKZF2* | -2.320 |
| *RNF144A* | -2.576 | *LINC00426* | -2.309 |
| *RP11-27K13.3* | -2.568 | *ATP8A1* | -2.307 |
| *GRM2* | -2.566 | *STMN1* | -2.297 |
| *ADC* | -2.562 | *CD101* | -2.295 |
| *AL358813.2* | -2.559 | *FAM111B* | -2.292 |
| *RP5-1139B12.2* | -2.558 | *LAIR1* | -2.291 |
| *CTA-833B7.2* | -2.556 | *H2AFY2* | -2.279 |
| *FRY* | -2.552 | *SGK223* | -2.275 |
| *MVB12B* | -2.549 | *ZNF300* | -2.268 |
| *MCTP1* | -2.549 | *CCL3L3* | -2.256 |
| *CTD-2175A23.1* | -2.545 | *TPM2* | -2.256 |
| *NBEAL2* | -2.545 | *CDCA3* | -2.251 |
| *HIP1* | -2.541 | *POLQ* | -2.248 |
| *TTC39B* | -2.534 | *CDKN2D* | -2.246 |
| *PITPNC1* | -2.532 | *CTC-523E23.11* | -2.243 |
| *XYLT1* | -2.532 | *KIAA1107* | -2.241 |
| *RAB37* | -2.528 | *ABLIM1* | -2.231 |
| *FAM65B* | -2.527 | *PLXNA4* | -2.225 |
| *IFIT2* | -2.527 | *RASSF8-AS1* | -2.225 |
| *RP11-1000B6.5* | -2.527 | *ST8SIA6* | -2.223 |
| *DNAH10OS* | -2.527 | *SHCBP1* | -2.214 |
| *RP11-763B22.3* | -2.527 | *PFN1P7* | -2.212 |
| *JMJD7-PLA2G4B* | -2.511 | *LINC00528* | -2.212 |
| *AC013264.2* | -2.510 | *PDGFD* | -2.212 |
| *YPEL3* | -2.508 | *FAM47E-STBD1* | -2.204 |
| *RP11-632K20.7* | -2.504 | *RIMBP3* | -2.204 |
| *ACACB* | -2.504 | *KIF4A* | -2.201 |
| *RP3-508I15.18* | -2.501 | *CTD-2587H19.1* | -2.200 |
| *ABCG1* | -2.501 | *CENPA* | -2.198 |
| *THBS1* | -2.500 | *ST8SIA1* | -2.198 |
| *C8orf46* | -2.500 | *AC009133.17* | -2.197 |
| *RP11-661A12.14* | -2.496 | *CLU* | -2.193 |
| *RP11-403P17.3* | -2.492 | *RAB37* | -2.186 |
| *ERMP1* | -2.482 | *ZGLP1* | -2.185 |
| *SAP25* | -2.481 | *AC016683.6* | -2.177 |
| *CCL5* | -2.478 | *CCDC109B* | -2.173 |
| *RP11-353N4.7* | -2.471 | *TMEM204* | -2.169 |
| *CCL3L1* | -2.468 | *AKR1C3* | -2.168 |
| *FKBP6* | -2.461 | *RP1-151B14.6* | -2.165 |
| *RP11-533E19.7* | -2.460 | *FRY* | -2.163 |
| *CHMP7* | -2.458 | *C8orf46* | -2.162 |
| *F2R* | -2.455 | *RP11-561C5.5* | -2.161 |
| *PLEC* | -2.450 | *RP11-18I14.10* | -2.158 |
| *RP11-4O1.2* | -2.444 | *CHN2* | -2.149 |
| *RP6-206I17.4* | -2.442 | *PTGDR2* | -2.148 |
| *RP11-458D21.1* | -2.441 | *RP11-867O8.5* | -2.148 |
| *RFPL2* | -2.441 | *NUAK2* | -2.142 |
| *CSGALNACT1* | -2.438 | *KIF2C* | -2.141 |
| *ABHD17AP3* | -2.437 | *AC118278.1* | -2.140 |
| *BBC3* | -2.436 | *RP11-421L21.3* | -2.139 |
| *ADAMTS10* | -2.436 | *RNF214* | -2.130 |
| *NTNG2* | -2.432 | *ANKRD32* | -2.130 |
| *LRFN1* | -2.428 | *RGS17* | -2.128 |
| *IL15* | -2.417 | *RGS18* | -2.128 |
| *RP11-495P10.10* | -2.409 | *CECR1* | -2.128 |
| *ABTB1* | -2.409 | *RNU1-143P* | -2.127 |
| *RP6-206I17.3* | -2.401 | *TRAM2* | -2.124 |
| *KIF18B* | -2.401 | *ZWINT* | -2.110 |
| *AF131217.1* | -2.394 | *ZNF541* | -2.102 |
| *AC000032.2* | -2.393 | *LRP5* | -2.090 |
| *KANK1* | -2.393 | *KCNA2* | -2.089 |
| *RP5-1184F4.5* | -2.382 | *ESCO2* | -2.085 |
| *PLEKHG3* | -2.381 | *ANK3* | -2.083 |
| *AMY1C* | -2.379 | *IL24* | -2.080 |
| *RP3-508I15.19* | -2.379 | *RP11-493L12.4* | -2.078 |
| *COL11A2* | -2.377 | *AC099522.1* | -2.078 |
| *AC007952.5* | -2.375 | *FOXD1* | -2.078 |
| *OSER1-AS1* | -2.373 | *CHI3L2* | -2.078 |
| *RAB27B* | -2.371 | *KANSL1-AS1* | -2.078 |
| *SLC44A5* | -2.362 | *LPAR6* | -2.077 |
| *CCL4L2* | -2.359 | *RP11-347I19.8* | -2.075 |
| *CDCA3* | -2.359 | *FAM47E* | -2.071 |
| *CCL4L1* | -2.348 | *APOBEC3H* | -2.065 |
| *MROH6* | -2.348 | *LTB* | -2.064 |
| *RP3-465N24.5* | -2.343 | *PLCD1* | -2.063 |
| *MIR3615* | -2.343 | *RRM2* | -2.059 |
| *PNMA3* | -2.339 | *AL358813.2* | -2.056 |
| *INADL* | -2.332 | *PLA2G4B* | -2.053 |
| *TTC21A* | -2.332 | *PFN1P6* | -2.053 |
| *HJURP* | -2.331 | *RP5-1092A3.4* | -2.049 |
| *GRIP1* | -2.329 | *ADARB2* | -2.043 |
| *RP11-403I13.8* | -2.319 | *GCNT1* | -2.042 |
| *ATP2A1* | -2.316 | *OGFRL1* | -2.041 |
| *SUN2* | -2.315 | *CAMK2G* | -2.041 |
| *ZMYND10* | -2.304 | *LINC00514* | -2.039 |
| *AMY1A* | -2.287 | *RP11-1000B6.5* | -2.039 |
| *RP11-347I19.8* | -2.284 | *AGBL3* | -2.037 |
| *HIST1H2AC* | -2.282 | *SPNS3* | -2.036 |
| *C16orf98* | -2.280 | *SIGIRR* | -2.033 |
| *CDCP1* | -2.268 | *AC147651.4* | -2.032 |
| *CTSF* | -2.266 | *E2F8* | -2.029 |
| *IFI27* | -2.264 | *SKA3* | -2.027 |
| *RP11-79D8.2* | -2.264 | *TPX2* | -2.018 |
| *RP11-160E2.19* | -2.264 | *LINC00086* | -2.016 |
| *AC093162.5* | -2.258 | *AKTIP* | -2.015 |
| *RP5-882C2.2* | -2.255 | *CYB561* | -2.004 |
| *AC092316.2* | -2.252 | *ADHFE1* | -2.003 |
| *YPEL1* | -2.250 | *CDCA2* | -2.003 |
| *NMUR1* | -2.246 | *SEPP1* | -2.002 |
| *DDX43* | -2.245 | *SLC2A14* | 2.000 |
| *SH3GL1P3* | -2.244 | *GFOD1* | 2.005 |
| *CSNK1G2-AS1* | -2.243 | *RP1-134E15.3* | 2.010 |
| *C1orf21* | -2.241 | *MIIP* | 2.010 |
| *GALNT8* | -2.237 | *CMA1* | 2.011 |
| *RASSF1* | -2.229 | *SYT15* | 2.013 |
| *ADHFE1* | -2.228 | *SYTL3* | 2.013 |
| *NLRP1* | -2.227 | *IFI30* | 2.016 |
| *AC017076.5* | -2.227 | *C17orf58* | 2.017 |
| *RP11-335G20.7* | -2.224 | *PPME1* | 2.018 |
| *RP4-594I10.3* | -2.223 | *LINC00263* | 2.018 |
| *XXcos-LUCA11.4* | -2.222 | *GPC1* | 2.020 |
| *CICP26* | -2.222 | *SBF2-AS1* | 2.022 |
| *PLEKHB1* | -2.220 | *AC009299.2* | 2.023 |
| *RP11-37C7.3* | -2.219 | *RP11-277E18.2* | 2.025 |
| *TLE1* | -2.218 | *RP11-277J24.1* | 2.025 |
| *RP3-508I15.22* | -2.213 | *RP11-815D16.1* | 2.025 |
| *RP3-508I15.20* | -2.203 | *MB21D2* | 2.029 |
| *RP11-89F3.2* | -2.202 | *NANOGP1* | 2.031 |
| *RGS17* | -2.198 | *STX11* | 2.035 |
| *PARP8* | -2.197 | *PGAM4* | 2.036 |
| *CTD-2288O8.1* | -2.194 | *ZNF215* | 2.038 |
| *EFCAB4A* | -2.194 | *TRPS1* | 2.038 |
| *RP11-75C10.7* | -2.191 | *ATP2A1* | 2.043 |
| *RP11-225H22.7* | -2.188 | *SETBP1* | 2.048 |
| *ATP8A1* | -2.186 | *OASL* | 2.051 |
| *RP11-1114A5.4* | -2.186 | *BLVRA* | 2.051 |
| *ADAM23* | -2.185 | *RP11-642P15.1* | 2.053 |
| *ZMAT1* | -2.183 | *GRAMD3* | 2.054 |
| *C17orf103* | -2.183 | *RP4-539M6.19* | 2.055 |
| *CTA-211A9.5* | -2.180 | *SVILP1* | 2.056 |
| *ARRDC5* | -2.179 | *AC092580.3* | 2.060 |
| *LINC00892* | -2.178 | *VTI1B* | 2.064 |
| *ERBB3* | -2.177 | *GAPDHP63* | 2.067 |
| *CD40LG* | -2.176 | *CSPG4P12* | 2.070 |
| *AC118278.1* | -2.174 | *RP11-158G18.1* | 2.071 |
| *LINC00528* | -2.170 | *RP5-836N10.1* | 2.073 |
| *AC099522.1* | -2.164 | *SLC39A8* | 2.076 |
| *FOXD1* | -2.164 | *CTSG* | 2.077 |
| *RP11-383J24.6* | -2.164 | *FLVCR2* | 2.085 |
| *OLFM2* | -2.160 | *COPG2* | 2.087 |
| *CAPG* | -2.156 | *PARPBP* | 2.089 |
| *RP11-353N4.4* | -2.155 | *MIR3918* | 2.092 |
| *SPNS3* | -2.155 | *RP13-608F4.5* | 2.092 |
| *LMF1* | -2.154 | *HIVEP1* | 2.094 |
| *SPTB* | -2.151 | *SLC2A3* | 2.095 |
| *ABHD17AP4* | -2.150 | *P4HA3* | 2.096 |
| *TGM1* | -2.150 | *CNTF* | 2.097 |
| *SYNE1* | -2.149 | *AC004540.4* | 2.100 |
| *AMPH* | -2.148 | *TFRC* | 2.101 |
| *H2AFY2* | -2.147 | *PPAN* | 2.103 |
| *RP11-1149O23.2* | -2.143 | *MIR155HG* | 2.106 |
| *LTB4R* | -2.138 | *TRABD2A* | 2.107 |
| *CTD-2207L17.2* | -2.138 | *MTFP1* | 2.113 |
| *SIX4* | -2.137 | *SCARB1* | 2.130 |
| *MYBL1* | -2.136 | *PMAIP1* | 2.137 |
| *KIF20A* | -2.134 | *RP11-1399P15.1* | 2.137 |
| *C5* | -2.132 | *PIM3* | 2.140 |
| *SUSD3* | -2.131 | *BCL2L1* | 2.142 |
| *CTD-2576D5.4* | -2.127 | *FABP5* | 2.143 |
| *FGD3* | -2.125 | *NCKIPSD* | 2.144 |
| *CALHM2* | -2.125 | *FAM167A* | 2.145 |
| *RP11-1136G11.8* | -2.125 | *SLC7A5* | 2.151 |
| *KLKB1* | -2.119 | *LINC00271* | 2.151 |
| *RASGEF1A* | -2.117 | *DEPDC7* | 2.152 |
| *C20orf197* | -2.112 | *IL18RAP* | 2.156 |
| *FGFBP2* | -2.112 | *RP11-104E19.1* | 2.158 |
| *TRIM17* | -2.112 | *SLC9A7* | 2.159 |
| *FAM47E-STBD1* | -2.112 | *NFIL3* | 2.161 |
| *LINC00623* | -2.107 | *GPR160* | 2.163 |
| *RP3-508I15.14* | -2.103 | *PAICS* | 2.167 |
| *C6ORF50* | -2.097 | *AMZ2P2* | 2.173 |
| *RNF44* | -2.097 | *ICAM1* | 2.176 |
| *ANKRD24* | -2.094 | *P2RX5-TAX1BP3* | 2.178 |
| *GAB3* | -2.091 | *ST7* | 2.179 |
| *PLA2G6* | -2.089 | *CISH* | 2.183 |
| *RP13-297E16.4* | -2.089 | *RP11-54F2.1* | 2.185 |
| *LAIR1* | -2.087 | *LDHAP2* | 2.192 |
| *WHAMMP3* | -2.085 | *SREBF1* | 2.196 |
| *RP6-206I17.2* | -2.084 | *RP4-536B24.2* | 2.196 |
| *C9orf156* | -2.079 | *SPINT1* | 2.196 |
| *NRBP2* | -2.078 | *STARD4-AS1* | 2.197 |
| *ABHD17AP5* | -2.076 | *STRIP2* | 2.202 |
| *MYH7B* | -2.076 | *RP11-10A14.3* | 2.203 |
| *CTD-2562J17.9* | -2.075 | *CCL3* | 2.204 |
| *S100A4* | -2.074 | *ZBTB10* | 2.212 |
| *RP11-493L12.4* | -2.073 | *SLC16A3* | 2.213 |
| *PRRC2B* | -2.073 | *AC093818.1* | 2.217 |
| *RP11-18H21.1* | -2.070 | *SQLE* | 2.231 |
| *CCDC109B* | -2.061 | *IPMK* | 2.233 |
| *RP11-353N4.6* | -2.056 | *MIR29B1* | 2.237 |
| *ANLN* | -2.056 | *FOXP3* | 2.237 |
| *AC104532.2* | -2.055 | *DUSP6* | 2.256 |
| *NCF4* | -2.050 | *DUSP1* | 2.257 |
| *C4B* | -2.050 | *FOSL2* | 2.257 |
| *LLGL2* | -2.049 | *RP11-84D1.1* | 2.258 |
| *RP11-640M9.1* | -2.047 | *CD86* | 2.262 |
| *RP11-62C7.2* | -2.046 | *RP11-152F13.7* | 2.263 |
| *RP11-284N8.3* | -2.046 | *IL3RA* | 2.270 |
| *SLC39A10* | -2.045 | *CSPG4P9* | 2.278 |
| *CIT* | -2.043 | *IL4R* | 2.280 |
| *TCEANC* | -2.043 | *ICAM5* | 2.288 |
| *RP11-75C10.9* | -2.043 | *BIRC3* | 2.289 |
| *SELPLG* | -2.043 | *AC006460.2* | 2.289 |
| *RPL32P1* | -2.041 | *RAVER2* | 2.289 |
| *KCNA6* | -2.041 | *PSAT1* | 2.299 |
| *STK38* | -2.039 | *JUNB* | 2.301 |
| *RP11-47I22.3* | -2.038 | *SPON1* | 2.304 |
| *RNU1-68P* | -2.036 | *P4HA2-AS1* | 2.304 |
| *Z85986.1* | -2.035 | *ICAM2* | 2.307 |
| *AC109826.1* | -2.034 | *ELL3* | 2.308 |
| *MAML2* | -2.031 | *SYP* | 2.316 |
| *RP11-666A1.3* | -2.029 | *TPI1P2* | 2.317 |
| *LIMD2* | -2.029 | *IL1RAP* | 2.320 |
| *CTD-3222D19.7* | -2.025 | *AC103965.1* | 2.328 |
| *GPT* | -2.024 | *C14orf182* | 2.333 |
| *AL358781.1* | -2.024 | *CTD-2377D24.6* | 2.337 |
| *RP13-977J11.2* | -2.023 | *RP11-295D4.1* | 2.337 |
| *TRIM3* | -2.022 | *C17orf72* | 2.339 |
| *TOX* | -2.021 | *BNIP3* | 2.340 |
| *THRA* | -2.018 | *AC013460.1* | 2.344 |
| *BAIAP3* | -2.014 | *PDE4D* | 2.345 |
| *DNASE1L2* | -2.012 | *ARHGAP31* | 2.347 |
| *CTD-2015B23.2* | -2.009 | *HMGB3P32* | 2.347 |
| *AC005071.1* | -2.005 | *AC007278.2* | 2.355 |
| *ITGA6* | -2.005 | *PHKA1* | 2.356 |
| *DUOX1* | -2.002 | *CAMK1* | 2.357 |
| *LINC01011* | -2.002 | *EPSTI1* | 2.363 |
| *DENND5A* | -2.000 | *RP11-549L6.3* | 2.367 |
| *RMI2* | 2.004 | *HAVCR1* | 2.370 |
| *HMGCS1* | 2.004 | *LAIR2* | 2.374 |
| *P2RX5* | 2.007 | *NAMPT* | 2.376 |
| *UBE2F* | 2.011 | *BCAT1* | 2.378 |
| *RP11-39C10.1* | 2.016 | *CD274* | 2.383 |
| *AC073046.25* | 2.021 | *PITPNM2* | 2.389 |
| *NME1-NME2* | 2.023 | *CLCF1* | 2.406 |
| *NINJ1* | 2.032 | *RP1-71H24.1* | 2.406 |
| *RP5-836N10.1* | 2.038 | *NDFIP2* | 2.408 |
| *NME2* | 2.042 | *RP11-561C5.4* | 2.411 |
| *BNIP3L* | 2.042 | *PFKFB3* | 2.412 |
| *PMAIP1* | 2.042 | *DBN1* | 2.413 |
| *NDFIP2* | 2.043 | *BATF* | 2.415 |
| *BZW1P1* | 2.044 | *MYOF* | 2.420 |
| *RP11-429G19.3* | 2.045 | *EGR3* | 2.423 |
| *PSMD14* | 2.054 | *AC007278.3* | 2.428 |
| *CTNS* | 2.057 | *CSPG4P8* | 2.429 |
| *LDHAP2* | 2.058 | *SH2D2A* | 2.447 |
| *RP1-134E15.3* | 2.058 | *DOK4* | 2.449 |
| *CORO1C* | 2.058 | *DUSP2* | 2.456 |
| *RP11-54F2.1* | 2.058 | *ENO1-IT1* | 2.457 |
| *DNM1P38* | 2.058 | *SERPINE2* | 2.458 |
| *AFAP1L2* | 2.062 | *RP11-152F13.3* | 2.459 |
| *SLC2A14* | 2.067 | *SMS* | 2.464 |
| *SBF2-AS1* | 2.069 | *IZUMO4* | 2.467 |
| *HMGB3P32* | 2.070 | *ANKRD33B* | 2.475 |
| *PHLDA1* | 2.073 | *NR4A2* | 2.478 |
| *SLC30A3* | 2.073 | *SLCO4A1* | 2.479 |
| *DOCK3* | 2.075 | *CSPG4P10* | 2.481 |
| *VLDLR* | 2.080 | *TCTN3* | 2.484 |
| *NT5DC2* | 2.083 | *DFNB31* | 2.485 |
| *RP11-298C3.2* | 2.090 | *OAS1* | 2.491 |
| *JAM3* | 2.091 | *CD9* | 2.493 |
| *CTB-120L21.1* | 2.091 | *AC017002.2* | 2.495 |
| *SLC2A3* | 2.091 | *NAMPTL* | 2.499 |
| *PANX1* | 2.091 | *RP13-996F3.3* | 2.504 |
| *TLE2* | 2.093 | *FBXO16* | 2.505 |
| *CRYBG3* | 2.093 | *LTA* | 2.506 |
| *CCR8* | 2.098 | *AEBP1* | 2.507 |
| *SLC35F3* | 2.103 | *CSF1* | 2.517 |
| *GZMH* | 2.113 | *MTHFD2* | 2.521 |
| *TPMT* | 2.120 | *AIM2* | 2.531 |
| *VDAC1P6* | 2.122 | *ZNF395* | 2.531 |
| *HSP90AB3P* | 2.123 | *RP11-28F1.2* | 2.563 |
| *RP11-274E7.2* | 2.126 | *ENO1* | 2.567 |
| *RP13-143G15.3* | 2.129 | *MX2* | 2.574 |
| *PLEKHN1* | 2.129 | *RSPH3* | 2.587 |
| *MIR3918* | 2.129 | *NFKBIZ* | 2.590 |
| *DTHD1* | 2.130 | *RP11-215G15.5* | 2.600 |
| *IRF5* | 2.132 | *RP11-324I22.3* | 2.603 |
| *IMMP2L* | 2.132 | *BCL2* | 2.607 |
| *HSPD1* | 2.138 | *MAL* | 2.615 |
| *AGRN* | 2.142 | *SLC43A3* | 2.621 |
| *ANKRD20A5P* | 2.146 | *ENPP3* | 2.623 |
| *DUSP3* | 2.146 | *ZNF629* | 2.623 |
| *RP5-940J5.9* | 2.151 | *RP6-99M1.2* | 2.630 |
| *FZD3* | 2.153 | *BNIP3P1* | 2.633 |
| *MB21D2* | 2.157 | *SOWAHA* | 2.638 |
| *YBX1P2* | 2.158 | *CAMKK1* | 2.645 |
| *CD68* | 2.166 | *P4HA2* | 2.648 |
| *KCNN4* | 2.172 | *ABTB2* | 2.650 |
| *FADS2* | 2.178 | *RP11-872D17.8* | 2.653 |
| *AHI1* | 2.178 | *SLC39A14* | 2.655 |
| *CHAC2* | 2.182 | *RP11-516A11.1* | 2.659 |
| *BOLA3* | 2.184 | *TNFSF13B* | 2.659 |
| *MCM6* | 2.184 | *RP11-312J18.5* | 2.668 |
| *RP11-290L1.3* | 2.184 | *SBF2* | 2.670 |
| *TIMD4* | 2.185 | *KLRD1* | 2.672 |
| *GAPDH* | 2.194 | *CD83* | 2.674 |
| *LINC00263* | 2.199 | *SDC4* | 2.697 |
| *RP11-312J18.5* | 2.213 | *RP13-143G15.3* | 2.698 |
| *MTFP1* | 2.213 | *GBP6* | 2.700 |
| *BATF* | 2.218 | *PGK1* | 2.705 |
| *NCR3LG1* | 2.219 | *MRC2* | 2.706 |
| *PSME2P2* | 2.220 | *PHLDA1* | 2.715 |
| *SYTL3* | 2.224 | *GPR153* | 2.722 |
| *GALE* | 2.225 | *KCNH4* | 2.730 |
| *BCL2A1* | 2.232 | *RP11-192H23.5* | 2.731 |
| *DTL* | 2.234 | *TRAF4* | 2.732 |
| *RP4-539M6.19* | 2.234 | *GBP1* | 2.736 |
| *VTI1B* | 2.243 | *SOCS1* | 2.747 |
| *PARPBP* | 2.245 | *AC144831.1* | 2.754 |
| *SCARB1* | 2.250 | *GLDC* | 2.778 |
| *RP11-395L14.17* | 2.253 | *SIGLEC12* | 2.780 |
| *RP11-54O7.14* | 2.255 | *RASGEF1B* | 2.784 |
| *DGCR5* | 2.255 | *AC078883.3* | 2.791 |
| *PHLDB1* | 2.260 | *NINJ1* | 2.820 |
| *WNT16* | 2.260 | *JAM3* | 2.823 |
| *RP13-131K19.6* | 2.261 | *CERCAM* | 2.825 |
| *PIP5K1B* | 2.262 | *IFNG* | 2.847 |
| *GAPDHP1* | 2.264 | *USP41* | 2.850 |
| *HSP90AB1* | 2.265 | *AC084117.3* | 2.851 |
| *AC004069.2* | 2.267 | *AC017002.1* | 2.864 |
| *SPINT2* | 2.271 | *PLAUR* | 2.886 |
| *AC004540.4* | 2.277 | *ZDHHC4P1* | 2.902 |
| *SLC16A1* | 2.286 | *PSD* | 2.911 |
| *PUS7* | 2.291 | *ZDHHC9* | 2.916 |
| *ICAM1* | 2.294 | *GLIS2* | 2.918 |
| *GPR56* | 2.298 | *BHLHE40-AS1* | 2.919 |
| *HNRNPA1P21* | 2.312 | *ZNF282* | 2.925 |
| *CCR1* | 2.312 | *CREM* | 2.927 |
| *SNHG15* | 2.315 | *ALDOC* | 2.935 |
| *TCTN3* | 2.315 | *GZMB* | 2.936 |
| *ENO1P1* | 2.318 | *LDHAP3* | 2.936 |
| *CTD-2369P2.8* | 2.320 | *PGK1P2* | 2.939 |
| *HSPE1* | 2.331 | *C12orf68* | 2.945 |
| *MAP7* | 2.344 | *BBS5* | 2.945 |
| *IL4R* | 2.346 | *HK2* | 2.947 |
| *BCL2* | 2.349 | *MYO1B* | 2.958 |
| *AC010761.9* | 2.353 | *LIMA1* | 2.959 |
| *CISH* | 2.363 | *MICAL2* | 2.961 |
| *RP13-98N21.2* | 2.372 | *LYST-AS1* | 2.967 |
| *IMPDH2* | 2.373 | *DDIT4* | 2.974 |
| *RP3-388E23.2* | 2.373 | *PRKCE* | 2.988 |
| *HNRNPA1P54* | 2.380 | *NPTX1* | 3.003 |
| *HSPD1P1* | 2.389 | *DNM1P38* | 3.017 |
| *MCM10* | 2.390 | *RP11-638I2.10* | 3.021 |
| *RP11-215G15.5* | 2.391 | *AC010761.10* | 3.028 |
| *PLAUR* | 2.394 | *ZBTB32* | 3.043 |
| *PPME1* | 2.401 | *ICOSLG* | 3.044 |
| *RP11-490H24.5* | 2.411 | *RP11-442H21.2* | 3.045 |
| *RP11-316P17.2* | 2.416 | *USP18* | 3.048 |
| *TRAF4* | 2.419 | *LDHA* | 3.051 |
| *RP11-52J3.2* | 2.423 | *TPI1* | 3.056 |
| *BCL2L1* | 2.428 | *TPI1P1* | 3.078 |
| *VDAC1P2* | 2.430 | *RP11-290L1.3* | 3.089 |
| *SERPINB8* | 2.435 | *RP11-357H14.17* | 3.092 |
| *RXRA* | 2.437 | *RP13-98N21.2* | 3.097 |
| *NFIL3* | 2.437 | *LDHAP4* | 3.108 |
| *PGAM1P8* | 2.443 | *OSM* | 3.120 |
| *RP11-638I2.10* | 2.444 | *C3* | 3.146 |
| *SMS* | 2.450 | *RP11-498C9.13* | 3.151 |
| *PPA1* | 2.452 | *CCR9* | 3.155 |
| *GAPDHP65* | 2.453 | *RP11-54O7.14* | 3.155 |
| *FAM162A* | 2.454 | *PKM* | 3.162 |
| *SLC17A9* | 2.455 | *GBE1* | 3.169 |
| *RP11-887P2.3* | 2.461 | *DTHD1* | 3.170 |
| *PGAM1* | 2.461 | *VLDLR* | 3.191 |
| *TPRG1* | 2.466 | *RP11-265D17.2* | 3.194 |
| *CAMKK1* | 2.469 | *PLB1* | 3.202 |
| *SORD* | 2.473 | *PAICSP4* | 3.208 |
| *ACTN1* | 2.478 | *C4orf32* | 3.208 |
| *DHCR24* | 2.479 | *RP11-465B22.3* | 3.223 |
| *PGAM4* | 2.484 | *SNX9* | 3.226 |
| *MYOF* | 2.485 | *GPR56* | 3.231 |
| *GBP1P1* | 2.488 | *N4BP3* | 3.247 |
| *NPM3* | 2.495 | *CORO1C* | 3.256 |
| *CYP51A1* | 2.502 | *SPOCK1* | 3.280 |
| *FOSL2* | 2.505 | *SPRY1* | 3.295 |
| *RP11-153M3.1* | 2.513 | *MX1* | 3.295 |
| *CXCL10* | 2.518 | *RP11-52J3.2* | 3.304 |
| *AC063976.7* | 2.518 | *RXRA* | 3.306 |
| *RP13-608F4.5* | 2.518 | *FAM57A* | 3.315 |
| *BCL2L11* | 2.522 | *AK4P1* | 3.332 |
| *LDLR* | 2.526 | *PHLDB1* | 3.342 |
| *RP11-618P17.4* | 2.533 | *IFNLR1* | 3.344 |
| *RAMP1* | 2.540 | *RP11-288L9.4* | 3.354 |
| *SNX9* | 2.541 | *LAG3* | 3.378 |
| *LRRD1* | 2.542 | *AC103563.9* | 3.382 |
| *GPI* | 2.548 | *PGBD4P1* | 3.382 |
| *GAPDHP63* | 2.549 | *SLC41A2* | 3.390 |
| *GRAMD3* | 2.550 | *VDR* | 3.407 |
| *SYP* | 2.550 | *TSKU* | 3.414 |
| *C14orf182* | 2.551 | *AP001610.5* | 3.415 |
| *RP11-161M6.2* | 2.553 | *P2RX5* | 3.421 |
| *ARHGEF40* | 2.555 | *EVI5* | 3.434 |
| *BATF3* | 2.558 | *IFI6* | 3.437 |
| *RP11-465B22.3* | 2.585 | *SHF* | 3.441 |
| *DFNB31* | 2.605 | *BEND5* | 3.461 |
| *TTYH2* | 2.606 | *IFI44* | 3.477 |
| *AC010761.10* | 2.610 | *RP11-344N10.4* | 3.492 |
| *TFRC* | 2.615 | *NAB2* | 3.513 |
| *CSF1* | 2.616 | *WARS* | 3.520 |
| *ZDHHC9* | 2.617 | *HIF1A-AS2* | 3.527 |
| *AFAP1* | 2.618 | *IRF4* | 3.531 |
| *RP11-549L6.3* | 2.621 | *NTRK2* | 3.536 |
| *SDC4* | 2.622 | *RP11-638I2.8* | 3.546 |
| *CMSS1* | 2.625 | *PMCH* | 3.566 |
| *HSP90AB2P* | 2.627 | *GPT2* | 3.572 |
| *MYC* | 2.631 | *ANKRD30BL* | 3.581 |
| *RP11-265D17.2* | 2.639 | *ZBED2* | 3.584 |
| *SVILP1* | 2.641 | *TMPRSS3* | 3.615 |
| *FAM57A* | 2.642 | *TRIB1* | 3.626 |
| *RP11-642P15.1* | 2.643 | *AC063976.7* | 3.632 |
| *GBP6* | 2.643 | *ANO7* | 3.634 |
| *HBEGF* | 2.643 | *ENPEP* | 3.635 |
| *PHKA1* | 2.688 | *GADD45B* | 3.636 |
| *ENTPD1-AS1* | 2.688 | *RP3-405J10.3* | 3.638 |
| *IFI30* | 2.701 | *FSCN1* | 3.645 |
| *CD83* | 2.703 | *GPR146* | 3.646 |
| *FADS1* | 2.714 | *CD38* | 3.658 |
| *EGR2* | 2.721 | *DUSP5* | 3.662 |
| *GPT2* | 2.721 | *RP11-568N6.1* | 3.683 |
| *ABTB2* | 2.750 | *MYB* | 3.688 |
| *MCM2* | 2.754 | *UST* | 3.695 |
| *RP11-243J16.7* | 2.767 | *SNORA14* | 3.740 |
| *MTHFD2* | 2.776 | *WNT16* | 3.741 |
| *SLC39A14* | 2.777 | *TIMD4* | 3.743 |
| *FOS* | 2.786 | *RP11-638I2.9* | 3.747 |
| *SLC41A2* | 2.790 | *DUSP4* | 3.756 |
| *NAPSA* | 2.795 | *RYR2* | 3.756 |
| *GADD45B* | 2.795 | *RP11-54O7.18* | 3.770 |
| *SLCO4A1* | 2.798 | *BATF3* | 3.784 |
| *TPI1P2* | 2.806 | *RAMP1* | 3.791 |
| *RP11-84D1.1* | 2.811 | *MIR663A* | 3.799 |
| *MICAL2* | 2.827 | *RP11-162J8.3* | 3.813 |
| *BHLHE40-AS1* | 2.835 | *METRNL* | 3.817 |
| *EAF2* | 2.845 | *AGRN* | 3.821 |
| *CNTF* | 2.847 | *MICALCL* | 3.823 |
| *SOCS3* | 2.849 | *C17orf96* | 3.826 |
| *AL160175.1* | 2.856 | *TRBV29-1* | 3.829 |
| *OSM* | 2.866 | *AK4P3* | 3.835 |
| *RP11-568N6.1* | 2.866 | *IL2RA* | 3.843 |
| *NAMPTL* | 2.875 | *PTGIR* | 3.848 |
| *MAL* | 2.882 | *HAPLN3* | 3.849 |
| *DEPDC7* | 2.889 | *DENND5B-AS1* | 3.866 |
| *NAMPT* | 2.896 | *RP11-138A9.2* | 3.892 |
| *PRDX4* | 2.903 | *FES* | 3.898 |
| *TNFRSF4* | 2.903 | *EGR1* | 3.919 |
| *RP11-456K23.1* | 2.911 | *RP11-449P15.1* | 3.922 |
| *BNIP3P1* | 2.913 | *AE000661.37* | 3.922 |
| *RP11-638I2.9* | 2.925 | *TLE2* | 3.926 |
| *WARS* | 2.946 | *ZC3H12C* | 3.946 |
| *ENO1* | 2.951 | *RP11-325F22.2* | 3.993 |
| *C4orf32* | 2.961 | *ARG2* | 4.010 |
| *P4HA2-AS1* | 2.965 | *AL139819.1* | 4.027 |
| *ENO1-IT1* | 2.972 | *HOMER2* | 4.054 |
| *ENTPD1* | 2.992 | *NCR3LG1* | 4.141 |
| *DUSP5* | 2.998 | *P4HA1* | 4.174 |
| *CLIC4* | 3.018 | *RP11-155G14.5* | 4.260 |
| *GBE1* | 3.019 | *SEMA4A* | 4.270 |
| *ZC3H12C* | 3.034 | *SCD* | 4.293 |
| *STARD4* | 3.040 | *IL5* | 4.294 |
| *PLA2G4A* | 3.041 | *DGCR5* | 4.404 |
| *TESC* | 3.042 | *TNFRSF4* | 4.409 |
| *DUSP6* | 3.050 | *PIK3R6* | 4.419 |
| *IFNG* | 3.051 | *MTHFD1L* | 4.456 |
| *PGK1* | 3.056 | *CCR7* | 4.459 |
| *DNAH11* | 3.058 | *RP11-344N10.2* | 4.460 |
| *AC103563.9* | 3.058 | *PTPN3* | 4.471 |
| *SETBP1* | 3.072 | *HAVCR2* | 4.478 |
| *PKM* | 3.077 | *SDK2* | 4.559 |
| *PSAT1* | 3.112 | *IER3* | 4.559 |
| *TMPRSS3* | 3.121 | *TNIP3* | 4.579 |
| *RP11-1399P15.1* | 3.125 | *RP11-536K7.5* | 4.579 |
| *CDC6* | 3.131 | *CSF2* | 4.594 |
| *CDCA7L* | 3.140 | *PYCR1* | 4.600 |
| *ENPEP* | 3.144 | *GBP1P1* | 4.605 |
| *ZBED2* | 3.150 | *IFIT1* | 4.680 |
| *SDK2* | 3.170 | *SOCS3* | 4.683 |
| *METTL1* | 3.174 | *IL12RB2* | 4.695 |
| *FOXP3* | 3.183 | *RGS16* | 4.717 |
| *MORN2* | 3.196 | *SIK1* | 4.730 |
| *LAIR2* | 3.196 | *FAM43A* | 4.780 |
| *RP11-104E19.1* | 3.197 | *XXbac-BPG252P9.10* | 4.805 |
| *RP6-99M1.2* | 3.197 | *NR4A1* | 4.833 |
| *SOCS1* | 3.199 | *CTLA4* | 4.835 |
| *LIMA1* | 3.213 | *SLC6A20* | 4.853 |
| *RP11-638I2.8* | 3.220 | *RP1-38C16.2* | 4.861 |
| *ALDOC* | 3.258 | *AFAP1* | 4.874 |
| *TPI1* | 3.262 | *NTRK1* | 4.934 |
| *PAICS* | 3.272 | *PKMP1* | 4.952 |
| *RP11-80A15.1* | 3.287 | *ADAMTS7* | 4.967 |
| *AIM2* | 3.312 | *C15orf48* | 5.041 |
| *PRKCE* | 3.321 | *RNU6-786P* | 5.112 |
| *MYB* | 3.325 | *VCAM1* | 5.189 |
| *HK2* | 3.328 | *PFKFB4* | 5.268 |
| *TPI1P1* | 3.337 | *SECTM1* | 5.280 |
| *PIK3R6* | 3.352 | *BCL2L14* | 5.315 |
| *BEND5* | 3.356 | *FOS* | 5.343 |
| *KIAA1467* | 3.361 | *HMGB1P17* | 5.376 |
| *SHF* | 3.378 | *RP11-155G14.6* | 5.379 |
| *UST* | 3.380 | *CTB-120L21.1* | 5.537 |
| *RP11-54O7.18* | 3.380 | *FOSB* | 5.550 |
| *VDR* | 3.382 | *RP11-1100L3.8* | 5.602 |
| *PTGS2* | 3.389 | *AK4* | 5.621 |
| *DDIT4* | 3.390 | *CCL1* | 5.640 |
| *NME1* | 3.394 | *LAMP3* | 5.670 |
| *ZNF215* | 3.425 | *RP1-261G23.7* | 5.824 |
| *MYO1B* | 3.428 | *MIR210HG* | 5.851 |
| *PAICSP4* | 3.432 | *CRTAM* | 5.883 |
| *AC005264.2* | 3.458 | *VEGFA* | 5.978 |
| *P4HA2* | 3.462 | *NR4A3* | 6.024 |
| *FABP5P7* | 3.466 | *SLC30A3* | 6.031 |
| *GZMB* | 3.468 | *HILPDA* | 6.128 |
| *CREM* | 3.504 | *PTGIS* | 6.151 |
| *KIAA1462* | 3.505 | *EBI3* | 6.161 |
| *LIN7A* | 3.518 | *RIMKLA* | 6.170 |
| *RP11-442H21.2* | 3.522 | *CTTN* | 6.226 |
| *AC017002.2* | 3.523 | *IRF8* | 6.280 |
| *FOSB* | 3.524 | *GNG4* | 6.300 |
| *RP3-405J10.3* | 3.587 | *IL13* | 6.480 |
| *RP11-192H23.5* | 3.600 | *EGR2* | 6.506 |
| *EVI5* | 3.621 | *DACT3* | 6.888 |
| *RGS16* | 3.626 | *TNFRSF8* | 6.932 |
| *LDHAP3* | 3.627 | *EPB41L4B* | 7.141 |
| *DUSP4* | 3.640 | *CXCL10* | 7.175 |
| *PTPN3* | 3.658 | *INSRR* | 7.235 |
| *CMA1* | 3.683 | *IFIT3* | 8.161 |
| *PKMP1* | 3.702 | *IL10* | 8.235 |
| *CRTAM* | 3.717 | *TNFRSF9* | 8.428 |
| *RP11-324I22.3* | 3.743 | *PPFIA4* | 9.084 |
| *GPR183* | 3.774 | *MFSD2A* | 9.117 |
| *SIK1* | 3.813 | *TNFRSF18* | 10.362 |
| *SOWAHA* | 3.831 |  |  |
| *LDHAP4* | 3.856 |  |  |
| *RYR2* | 3.866 |  |  |
| *INSRR* | 3.866 |  |  |
| *CTSG* | 3.883 |  |  |
| *SLC29A1* | 3.891 |  |  |
| *HIF1A-AS2* | 3.916 |  |  |
| *IRF4* | 3.931 |  |  |
| *IER3* | 3.941 |  |  |
| *LDHA* | 3.942 |  |  |
| *LINC00271* | 3.962 |  |  |
| *C15orf48* | 3.965 |  |  |
| *SPRY1* | 3.970 |  |  |
| *P4HA1* | 4.022 |  |  |
| *ADAMTS7* | 4.065 |  |  |
| *AC084117.3* | 4.077 |  |  |
| *MTHFD1L* | 4.121 |  |  |
| *C17orf96* | 4.148 |  |  |
| *SNORA14* | 4.174 |  |  |
| *LMNA* | 4.179 |  |  |
| *IL2RA* | 4.186 |  |  |
| *AL139819.1* | 4.203 |  |  |
| *TNIP3* | 4.206 |  |  |
| *NTRK2* | 4.207 |  |  |
| *PLB1* | 4.228 |  |  |
| *RP11-344N10.4* | 4.251 |  |  |
| *RP11-344N10.2* | 4.260 |  |  |
| *SLC43A3* | 4.311 |  |  |
| *RP11-248J23.7* | 4.335 |  |  |
| *RP11-872D17.8* | 4.401 |  |  |
| *PTGIS* | 4.409 |  |  |
| *IL10* | 4.416 |  |  |
| *ZBTB32* | 4.430 |  |  |
| *RP11-536K7.5* | 4.448 |  |  |
| *PMCH* | 4.475 |  |  |
| *NR4A3* | 4.476 |  |  |
| *ARG2* | 4.480 |  |  |
| *SCD* | 4.499 |  |  |
| *EPB41L4B* | 4.545 |  |  |
| *GNA15* | 4.576 |  |  |
| *LTA* | 4.598 |  |  |
| *AK4P1* | 4.626 |  |  |
| *NPTX1* | 4.628 |  |  |
| *XXbac-BPG252P9.10* | 4.631 |  |  |
| *MIR210HG* | 4.731 |  |  |
| *GNG4* | 4.733 |  |  |
| *ANO7* | 4.748 |  |  |
| *BCAT1* | 4.777 |  |  |
| *FABP5* | 4.807 |  |  |
| *CTLA4* | 4.844 |  |  |
| *NTRK1* | 4.854 |  |  |
| *DENND5B-AS1* | 4.859 |  |  |
| *TRIB1* | 4.867 |  |  |
| *HOMER2* | 4.870 |  |  |
| *AK4P3* | 4.916 |  |  |
| *SLC6A20* | 4.965 |  |  |
| *MIR155HG* | 4.966 |  |  |
| *AC017002.1* | 4.976 |  |  |
| *CSF2* | 4.989 |  |  |
| *SIGLEC12* | 5.036 |  |  |
| *FSCN1* | 5.110 |  |  |
| *VEGFA* | 5.141 |  |  |
| *LAMP3* | 5.146 |  |  |
| *TSKU* | 5.146 |  |  |
| *RNU6-786P* | 5.167 |  |  |
| *IRF8* | 5.198 |  |  |
| *AK4* | 5.202 |  |  |
| *SEMA4A* | 5.418 |  |  |
| *PYCR1* | 5.445 |  |  |
| *IL21R-AS1* | 5.501 |  |  |
| *GINS2* | 5.654 |  |  |
| *VCAM1* | 5.717 |  |  |
| *TNFRSF18* | 5.826 |  |  |
| *RP1-38C16.2* | 5.959 |  |  |
| *RP1-261G23.7* | 6.097 |  |  |
| *IL21R* | 6.186 |  |  |
| *CTTN* | 6.208 |  |  |
| *CCL1* | 6.271 |  |  |
| *EBI3* | 6.284 |  |  |
| *FABP5P1* | 6.306 |  |  |
| *ADM* | 6.331 |  |  |
| *MFSD2A* | 6.380 |  |  |
| *RIMKLA* | 6.468 |  |  |
| *RP11-155G14.6* | 6.493 |  |  |
| *HILPDA* | 6.655 |  |  |
| *HLF* | 6.709 |  |  |
| *CCR7* | 6.724 |  |  |
| *PFKFB4* | 6.728 |  |  |
| *IL5* | 6.738 |  |  |
| *TNFRSF8* | 6.840 |  |  |
| *RP11-155G14.5* | 7.344 |  |  |
| *PTGIR* | 7.522 |  |  |
| *PPFIA4* | 7.673 |  |  |
| *DACT3* | 7.843 |  |  |
| *IL13* | 7.958 |  |  |
| *HMGB1P17* | 8.141 |  |  |
| *RP11-498C9.13* | 8.203 |  |  |
| *TNFRSF9* | 8.490 |  |  |
